# TREM-1 and TREM-2 Expression on CD14^+^ Cells in Bronchoalveolar Lavage Fluid in Pulmonary Sarcoidosis and Hypersensitivity Pneumonitis in the Context of T Cell Immune Response

**DOI:** 10.1155/2020/9501617

**Published:** 2020-05-14

**Authors:** M. Suchankova, J. Urban, M. Ganovska, E. Tibenska, K. Szaboova, E. Tedlova, F. Sandor, I. Majer, M. Bobovcak, I. Jonner, B. Konig, M. Bucova

**Affiliations:** ^1^Institute of Immunology, Faculty of Medicine Comenius University, Bratislava, Slovakia; ^2^National Institute for Tuberculosis, Lung Diseases and Thoracic Surgery, Vysne Hagy, Slovakia; ^3^Medirex Ltd., Bratislava, Slovakia; ^4^Department of Pneumology and Phthisiology, Faculty of Medicine Comenius University and University Hospital, Bratislava, Slovakia; ^5^Beckman Coulter Ltd., Slovakia; ^6^University of Economics in Bratislava, Faculty of Economic Informatics, Department of Operations Research and Econometrics, Bratislava, Slovakia; ^7^Institute of Economic Research of Slovak Academy of Sciences, Bratislava, Slovakia

## Abstract

**Background:**

Sarcoidosis and hypersensitivity pneumonitis (HP) are immunologically mediated processes caused by hypersensitivity reaction accompanied by similar features including lymphocytic alveolitis and granuloma formation. Recent studies describe the role of TREM receptors in T cell activation, differentiation, and granuloma formation. Alveolar macrophages activation via TREM receptors may be the key factor mediating subsequent immune response. The aim of the study was to analyse TREM-1 and TREM-2 expression to identify further molecular mechanisms participating in the immunopathogenesis of sarcoidosis and HP.

**Methods:**

Flow cytometry was performed to analyse TREM-1 and TREM-2 expression on CD14^+^ cells in bronchoalveolar lavage fluid from patients having sarcoidosis or HP and a control group.

**Results:**

The study proved increased TREM-1 expression on alveolar macrophages in pulmonary sarcoidosis and diminished TREM-1 expression in HP-Sarcoidosis: median: 76.7; HP: median: 29.9; control: median: 53.3, (sarcoidosis versus HP: *p* < 0.001; sarcoidosis versus control: *p* < 0.05). TREM-2 expression was increased in both, sarcoidosis and HP-sarcoidosis: median: 34.79; HP: median: 36.00; control: median: 12.98, (sarcoidosis versus control: *p* < 0.05; HP versus control: *p* < 0.05). Correlation analysis showed negative correlation between TREM-1 and total number of CD8^+^ cytotoxic T cells. In sarcoidosis TREM-1 expression decreased with changes of HRCT image, decrease in CD4/CD8 ratio and decrease in DLCO.

**Conclusions:**

Differences in TREM receptor expression in sarcoidosis (increase in TREM-1 and TREM-2) and HP (increase in TREM-2) and correlation analysis suggests that activation via TREM may participate in typical immunological characteristics of sarcoidosis and HP.

## 1. Introduction

Sarcoidosis and hypersensitivity pneumonitis (HP) are classified within diffuse parenchymal lung diseases [1]. Sarcoidosis is an idiopathic multisystem disease characterized by the development of noncaseating well-formed granulomas in various tissues in almost any organ system [2]. Hypersensitivity pneumonitis is an inflammatory process associated with repeated inhalation of known organic antigens or low-molecular-weight organic molecules leading to the development of poorly formed small granulomas in the small airways and interstitium [3]. Both sarcoidosis and HP are thought to be caused by an interaction of genetic susceptibility with a hypersensitivity reaction to environmental antigens. Immunologically mediated processes in these two diagnoses have some similar features (lymphocytic alveolitis, granuloma formation, type IV hypersensitivity). However, qualitative and quantitative immunological differences exist between sarcoidosis and HP (Table 1) [1–4]. The reason for these differences is not yet entirely clear.

Alveolar macrophages are the first cells in alveolar spaces retaining antigen [5]. The initial activation of alveolar macrophages is the key factor in driving of subsequent immune response. Triggering receptors expressed on myeloid cells (TREM) have a role in macrophage activation.

TREM-1 amplifies inflammation by the increase in inflammatory cytokines production (e.g., TNF-*α*, IL-1, and IL-6) through synergism with TLR (toll-like receptors) and NLR (nod-like receptors) signalling [6–9]. Recent studies also suggest an important role of the TREM-1 in initiating the adaptive immune response. TREM-1-signalling supports CD4^+^ T cells activation, homing of T cells to secondary lymphoid organs [10], and skew T cell response to the Th1 or Th17 direction [11]. Zangi et al. [12] described the unique MHC-dependent, a perforin-based killing mechanism involving activation of TLR7 and signalling through TREM-1 leading to CD8^+^ T cell deletion. Thus, the elevation in CD4^+^ T cell proliferation and the deletion of CD8^+^ clones via TREM-1 activation could subsequently participate in an increase in the CD4/CD8 T cell ratio at the site of inflammation.

The TREM-2/DAP12-mediated signalling promotes phagocytosis and dampens TLR signalling leading to decreased production of pro-inflammatory cytokines [13, 14]. In this regard, activation of TREM-2 has an opposing effect to TREM-1 and blocks the development of systemic inflammation. Its role in the induction of CD8^+^ T cell activation as well as the differentiation of CD4^+^ T cells is not yet clear. TREM-2 promotes some CD4^+^ T cell response mechanisms by upregulation of some molecules, such as MHC class II, CD40, and CD86, promotes survival of dendritic cells (DCs) and upregulation of CCR7 [15]. TREM-2/DAP12-mediated maturation of antigen-presenting cells (APC) may promote T cell proliferation and stimulation of T cell cytokine production with an impact on T cell IFN-*γ* production [16]. TREM2/DAP12 mediated signalling is involved in modulating the expression of several macrophage-associated genes, including those encoding known mediators of macrophage fusion, such as DC-STAMP and cadherin-1. TREM-2/DAP12 signalling is required for the cytokine-induced formation of giant cells and potentiates macrophage fusion. The knockdown of TREM-2 leads to severely decreased macrophage fusion, so the TREM-2 receptor appears to play a dominant role during macrophage fusion [17].

The above studies demonstrated the effect of TREM mediated activation on the expression of other molecules on the surface of antigen-presenting cells and the production of mediators that are associated with T cell activation and other immune mechanisms (e.g., granuloma formation). Differences in alveolar macrophage activation via TREM receptors in pulmonary sarcoidosis and HP may be critical in the subsequent activation of the T cell immune response and could participate in the well-known qualitative as well as quantitative differences in T cell activation between these disease entities.

The presented study compares TREM-1 and TREM-2 expression on alveolar macrophages in BAL fluid in patients with pulmonary sarcoidosis and HP. In the context of the recently proved relationship between TREM and T cell immune response, our study focuses on correlation analysis between the TREM receptors and T cell subsets. The subsequent correlation analysis includes the relationship between TREM receptors and results from routinely used diagnostic procedures DLCO (diffusing capacity of lungs for carbon monoxide) and acquisition of HRCT (high-resolution computed tomography) imaging of lungs.

## 2. Study Group and Methods

The study group consisted of 144 patients with sarcoidosis and 18 patients with hypersensitivity pneumonitis. Patients indicated to the bronchoalveolar lavage procedure without proved DPLD or other diagnoses with an impact on lung parenchyma were selected to the control group (CG). The control group (CG) involved 11 subjects with negative findings in bronchoalveolar lavage fluid, without clinical and radiological evidence of interstitial lung process. The diagnosis of sarcoidosis or HP was established in compliance with current guidelines published in the following documents: Sarcoidosis: *ATS/ERS/WASOG statement on sarcoidosis* [2]. HP: *Hypersensitivity Pneumonitis: Perspectives in Diagnosis and Management* [18].

The characteristics of each study group and the baseline immunologic characteristics from BALF in the context of T cell response in pulmonary sarcoidosis and HP are presented in Table 2.

The patients with pulmonary sarcoidosis (stage I and II) were subdivided into four subgroups according to the type of dominant HRCT signs: stage I–Ia and Ib, stage II–IIa and IIb. HRCT images were staged from Ia to IIb type using the following criteria: Ia-hilar and mediastinal lymphadenopathy with no pulmonary infiltrates; Ib-hilar and mediastinal lymphadenopathy+solitary nodules; IIa-hilar and mediastinal lymfadenopathy+numerous small pulmonary nodules building clusters in typical areas for sarcoidosis; IIb-hilar and mediastinal lymphadenopathy+pulmonary infiltrates or ground glass opacity. The subgroups were assessed by two independent observers—expert in radiology and pneumology, with no knowledge of the TREM expression results.

Bronchoscopy with bronchoalveolar lavage procedure was carried out at the National Institute for Tuberculosis, Lung Diseases and Thoracic Surgery, Vysne Hagy, Slovakia. Bronchoalveolar lavage fluid (BALF) harvesting was performed by instillation of 120 ml (in three successive 40 ml aliquots) of sterile normal saline into the right middle lobe and aspirated by gentle suction using a flexible fiberoptic bronchoscope. BALF was first filtered through a double layer of sterile gauze, centrifuged at 300 g for 15 min at 10°C, and supernatants were collected. Finally, the pelleted cells were used for fluorescence-activated cell sorting (FACS). FACS data were acquired using NAVIOS Flow Cytometer (Beckman Coulter France S.A.S) set to capture all events. The anti-CD14, anti-TREM-1, and anti-TREM-2 antibodies conjugated by phycoerythrin-cyanine7 conjugate (PC7; Beckman Coulter France S.A.S), phycoerythrin (PE; R&D Systems, Minneapolis, MN, USA), and allophycocyanin (APC; R&D Systems, Minneapolis, MN, USA), respectively, were used for the analysis of TREM receptors. The analysis that we used in our previous study [19] was performed in compliance with Flow Cytometry Protocol recommended by the manufacturer. Lymphocytes and lymphocyte subsets were measured using tetraCHROME CD45-FITC/CD4-PE/CD8-ECD/CD3-PC5 Antibody Cocktail (Beckman Coulter France S.A.S). FACS data were analysed using KALUZA software (Beckman Coulter France S.A.S).

TREM-1 and TREM-2 expression is presented as the percentage of TREM-1 and TREM-2 positive cells out of all CD14^+^ cells and mean fluorescence intensity (MFI) of 10,000 cells in BALF. Samples of subjects with autofluorescence activity were excluded from the study. CD3, CD4, and CD8 expression is presented as a percentage and total number of cells. The total number of cells (CD3, CD4, CD8) was calculated from the absolute count of BALF cells evaluated by flow cytometer (absolute count of cells per milliliter was obtained of filtered, not concentrated, not centrifuged BALF by Flow-Count Fluorospheres (Beckman Coulter Inc.)).

The study was approved by the Ethical Committee of Faculty of Medicine Comenius University in Bratislava and Ethical Committee of National Institute for Tuberculosis, Lung Diseases and Thoracic Surgery, Vysne Hagy. Written informed consent was obtained from all patients and control subjects.

## 3. Statistical Analysis

The one-sample Kolmogorov-Smirnov test was used to determine whether the investigated population followed a normal distribution. Nonparametric analysis of variance (Kruskal-Wallis) with Dunn posttest was used to determine the differences and statistical significance. The results were expressed as the median and interquartile range (IQR). Correlation analysis was performed by Spearman test. Multinomial ordered logit was used to prove the relationship between ordered dependent variable with categories Ia, Ib, IIa, IIb and selected independent variables. *P* value <0.05 was considered to indicate statistical significance. Statistical analysis was performed using SAS and Stata softwares.

## 4. Results

### 4.1. Increased TREM-1 Expression on Alveolar CD14^+^ Cells in Patients with Pulmonary Sarcoidosis

**Figure 1:**
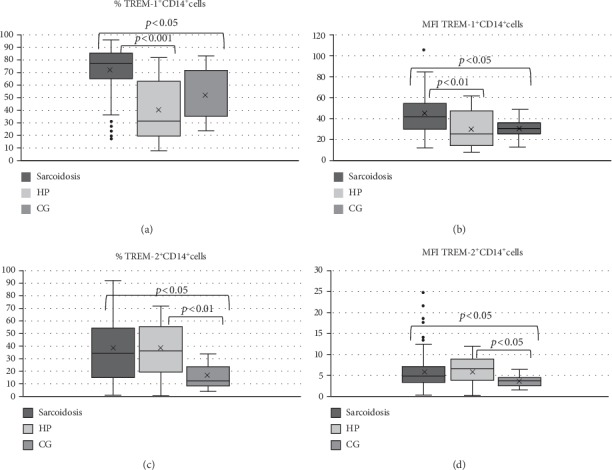
Comparison of TREM-1 and TREM-2 expression. (a) Percentage of TREM-1^+^CD14^+^ cells, (b) TREM-1 MFI, (c) percentage of TREM-2^+^ CD14^+^ cells, (d) TREM-2 MFI. HP: hypersensitivity pneumonitis; CG: control group.

In patients with pulmonary sarcoidosis we detected an increased percentage of TREM-1^+^ CD14^+^ cells and MFI compared with HP patients and CG subjects in BALF: percentage (Figure 1(a))—sarcoidosis: median: 76.7, IQR: 21.2; HP: median: 29.9, IQR: 43.6; CG: median: 53.3, IQR: 35.89 (sarcoidosis versus HP: *P* < 0.001; sarcoidosis versus CG: *P* < 0.05). MFI (Figure 1(b)): sarcoidosis: median: 40.67, IQR: 23.24; HP: median: 25.29, IQR: 33.7; CG: median: 30.53, IQR: 11.01 (sarcoidosis versus HP: *P* < 0.01; sarcoidosis versus CG *P* < 0.05). No significant difference in TREM-1 expression on CD14^+^ cells was found between HP and CG.

### 4.2. Increased TREM-2 Expression on Alveolar CD14^+^ Cells in Patients with Pulmonary Sarcoidosis and HP

TREM-2 expression on CD14^+^ cells from BALF was increased in pulmonary sarcoidosis and HP patients compared with CG subjects in both presented variables: percentage of TREM-2^+^ CD14^+^ cells (Figure 1(c))—sarcoidosis: median: 34.79, IQR: 38.93; HP: median: 36.00, IQR: 26.04; CG: median: 12.98, IQR: 16.06 (sarcoidosis versus CG: *P* < 0.05; HP versus CG: *P* < 0.05) and MFI (Figure 1(d))—sarcoidosis: median: 4.68, IQR: 3.89; HP: median: 6.9, IQR: 4.76; CG: median: 3.62, IQR: 2.22 (sarcoidosis versus CG: *P* < 0.05; HP versus CG: *P* < 0.05). No significant difference in TREM-2 expression on CD14^+^ BALF cells was found between sarcoidosis and HP patients.

### 4.3. Differences between TREM-1 and TREM-2 Expression on CD14^+^ Cells in Defined Clinical Subgroups in Sarcoidosis and HP

Patients with pulmonary sarcoidosis were subdivided according to the radiological stage (I/II/III/IV) and clinical presentation (with and without Lӧfgren syndrome) (Table 2). We found significantly higher TREM-1 expression (percentage of TREM-1^+^ cells) in stage I compared with stage II in sarcoidosis (*P* = 0.005; Table 3). Interestingly, the percentage of TREM-1^+^ cells in stage II of sarcoidosis was still significantly increased compared with HP (stage II sarcoidosis: median: 73.1, IQR: 20.2; HP: median: 29.9, IQR: 43.6; *P* = 0.0006). TREM-2 expression in both percentage and MFI (stage I versus stage II) variables was close to the specified level of statistical significance (*P* = 0.0648 or *P* = 0.0654, respectively). We did not find any significant differences in TREM-1 and TREM-2 expression in patients with and without Lӧfgren syndrome (*P* > 0.05; Table 3). Patients with HP were subdivided according to clinical presentation (inflammatory/fibrotic) (Table 2). We did not find any significant differences in TREM-1 and TREM-2 expression between the inflammatory versus fibrotic stage of the disease (*P* > 0.05; Table 3). In the end, we compared the inflammatory stages of sarcoidosis (stage I and II) with the inflammatory HP subgroup. We found still much higher TREM-1 expression in sarcoidosis compared with HP and the difference was statistically significant (percentage of TREM-1^+^CD14^+^ cells: *P* = 0.0002; TREM-1 MFI *P* = 0.0395; Table 3).

### 4.4. TREM-1 Expression on Alveolar CD14^+^ Cells Negatively Correlates with the Number of CD8^+^ T Cells and Positively Correlates with the Relative Portion of CD4^+^ T Cells as well as the CD4/CD8 Ratio

A correlation analysis was computed to assess the relationship between TREM expression, T cells, and T cell subsets. We found a strong negative correlation between TREM-1 expression and the total number and the relative portion of CD8^+^ cells and positive correlation between TREM-1 and the relative portion of CD4^+^ T cells (Table 4). We did not find any significant correlation between TREM-2 expression and T cell subsets.

### 4.5. DLCO (Diffusing Capacity of Lungs for Carbon Monoxide) Positively Correlates with TREM-1 Expression and the Relative Portion of CD4^+^ T Cells; Yet Negatively Correlates with CD8^+^ T Cell Subpopulation

DLCO is positively correlated with the percentage and MFI of TREM-1^+^ CD14^+^ cells and the relative portion of CD4^+^ T cells. In contrast, DLCO correlates negatively with the total number and the relative portion of CD8^+^ T cells (Table 5).

### 4.6. In Pulmonary Sarcoidosis, Percentage of TREM-1^+^ CD14^+^ Cells and CD4^+^ T Cells Decrease and Percentage of CD8^+^ T Cells Increases with HRCT Images Acquired from the most Benign to the most Severe Type

**Figure 2:**
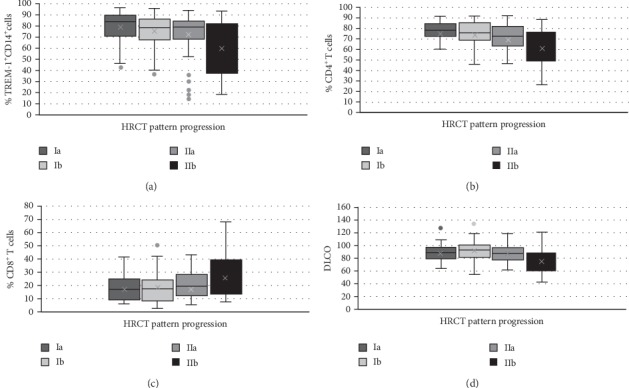
(a) Decrease in percentage of TREM-1^+^ CD14^+^ cells. (b) Decrease in percentage of CD4^+^ T cells. (c) Increase in percentage of CD8^+^ T cells. (d) Decrease in diffusing capacity of lungs for carbon monoxide (DLCO) in relationship with HRCT changes acquired from type Ia to type IIb. Ia-hilar and mediastinal lymphadenopathy with no pulmonary infiltrates; Ib-hilar and mediastinal lymphadenopathy and solitary nodules; IIa-hilar and mediastinal lymfadenopathy and numerous small pulmonary nodules building clusters in typical areas for sarcoidosis; IIb-hilar and mediastinal lymphadenopathy and pulmonary infiltrates or ground glass opacity.

We found that HRCT image acquired from type Ia to type IIb was accompanied by the decrease in the percentage of TREM-1^+^ CD14^+^ cells (Figure 2(a)), CD4^+^ T cells (Figure 2(b)) and DLCO (Figure 2(d)) and increase in CD8^+^ T cells (Figure 2(c)). These results are supported by the negative/positive coefficients of TREM-1^+^, CD4^+^, DLCO, CD8^+^ meaning that one unit increase in these variables decrease/increase the log odds of having the higher type of HRCT image, and thus higher levels of these variables lead to lower/higher stages of HRCT image (Table 6) (For the purpose of higher robustness of these findings we extended the multinomial ordered logit models in Table 6 by the other factors (TREM-1%, CD4^+^ T cells %, CD8^+^ T cells %, DLCO, TREM-2%, smokers, age, macrophages %, eosinophiles %, neutrophils %, lymphocytes %, lymphocytes total, sACE) that may affect HRCT image. These results support our conclusions achieved in Table 6 (see Table 7) that decrease in TREM-1^+^, CD4^+^, DLCO and increase in CD8^+^ is associated with worsening of HRCT image, even if we control for other factors.).

## 5. Discussion

Our results suggest that in cases of pulmonary sarcoidosis increase in TREM-1 expression could lead to its activation and increased production of proinflammatory cytokines promoting the development of systemic inflammation and typical systemic symptoms such as fever, weakness, and lymphadenopathy. In addition, upon TREM-1 activation, expression of the chemokine receptors CXCR4, CCR7, and costimulatory molecules CD83 and CD86 occur [10], allowing APC to enter the lymph nodes and promote antigen presentation to T helper cells. TREM-2 signalling is required for the formation of giant cells and potentiation of macrophage fusion [17] related to the formation of granulomas, a typical feature of the disease.

Our team has found that in cases of HP, the TREM-1 expression was significantly decreased compared with sarcoidosis. We hypothesize that the genetic predisposition or different character of antigens may be the cause of low expression of TREM-1. In our study, the TREM-1 expression could not be compared according to the type of the antigen due to the low number of subjects with HP, the various spectrum of exposing antigens and partially not clearly defined antigen in our study group (Table 2). Further studies, considering antigen exposure in HP, are needed for better understanding of alveolar macrophages activation via TREM-1. However, from our data, we could suggest that the low expression of TREM-1 can result in low proinflammatory cytokine production, limited development of systemic inflammation, and localization of the pathological process intrapulmonary. The low expression of TREM-1 may also have an impact, via the amount and spectra of proinflammatory cytokines production, to the granuloma formation leading to poorly defined small granulomas in HP compared with sarcoidosis. Increased expression of TREM-2 may be involved in macrophage fusion and granuloma formation by an increase in expression of mediators of macrophage fusion, such as DC-STAMP and cadherin-1 similar to sarcoidosis.

Our results of correlation analysis suggest a link between TREM-1 expression and T cell immune response. Our data are thus consistent with recently published studies showing this association *in vitro* [11, 12]. We found a strong negative correlation between TREM-1 and the total number of CD8^+^ T cells. Elevated expression of TREM-1 in pulmonary sarcoidosis can contribute to CD8^+^ T cell deletion by TREM-1 dependent perforin-granzyme mechanism. As a consequence of CD8^+^ T cell deletion, CD4^+^ T cells relatively predominate and the CD4/CD8 ratio is increased. In HP, low TREM-1 expression restricted CD8^+^ deletion resulting in higher T cell counts and normal or reduced CD4/CD8 ratio. CD4^+^ T cells positively correlated with TREM-1 only in a relative proportion, but not in the total numbers, suggesting that a positive correlation is related to CD8^+^ T cell deletion rather than an increase in the total number of CD4^+^ T cells. However, the detailed cellular and molecular mechanisms in sarcoidosis and HP are not yet known and require further research.

The results of TREM receptor expression were further correlated with clinically available data. We found a positive correlation between DLCO and TREM-1. We also noted a negative correlation between the DLCO and total number of CD8^+^ T cells (with impact on CD4/CD8 ratio). In pulmonary sarcoidosis, we noticed a decrease in TREM-1^+^CD14^+^ cells, CD4^+^ T cells, DLCO and increase in CD8^+^ T cells in relationship with HRCT images acquired from the most benign to the most severe type. The above findings show that a decrease in expression of TREM-1 accompanied by an increase in CD8^+^ T cells and a decrease in CD4/CD8 ratio appears to be associated with disease progression and worsening of gas exchange across the alveolar–capillary membrane. Some studies suggest the role of CD8^+^ T cells in the fibrotic process [20, 21]. Yet the specific role of TREM-1 (or absence of TREM-1 expression) in the chronic/fibrotic process is currently not known and requires further scientific investigation.

## 6. Conclusion

Our results show differences in TREM-1 and TREM-2 expression on CD14^+^ cells in bronchoalveolar lavage between pulmonary sarcoidosis and HP. Recent studies demonstrate the role of TREM receptors in the T cell immune response, the involvement of TREM-1 in the activation of systemic inflammation, and the role of TREM-2 in granuloma formation. Our study suggests that the alveolar macrophages activation via TREM receptors could participate in the typical immunological features of sarcoidosis and HP.

## Figures and Tables

**Table 1:** Differences in immunological features between sarcoidosis and HP.

	Sarcoidosis	Hypersensitivity pneumonitis
Total number of cells and percentage of lymphocytes in BALF	Normal or mildly elevated total cell count with lymphocytosis (usually <50% of lymphocytes)	High total cell count (usually >20 × 10^6^/100 mL BALF) with remarkable increment of lymphocytes (usually >50%)
CD4/CD8 ratio in BALF	Tendency to be increased	Tendency to be decreased/normal (varies according to the type of antigen, the intensity of exposure, smoking habit...)
Granulomas	Well-formed, tightly packed	Poorly formed small
Site of pathologic process	Perilymphatic, peribronchovascular, interstitium	Centrilobular, bronchiolocentric and peribronchiolal interstitium
Lymphadenopathy	Mediastinal and hilar/generalized	No/mediastinal and hilar may be present
Splenomegaly	Yes/no	No
Multisystem disorder	Yes	No

**Table 2:** Characteristics of the study group.

	Sarcoidosis	HP	CG
Number of subjects	144	18	11
Age (mean)	46	49	52
Gender: female (%)/male (%)	28/72	41/59	36/64
Smokers (%)/ex-smokers (%)/non-smokers (%)	6/22/74	6/33/61	9/9/82
Stage: I/II/III/IV (%) (sarcoidosis)	45/52/2/1		
Lӧfgren syndrome: Yes/no (%)	29/71		
Clinical presentation: Inflammatory/fibrotic (%) (HP)		57/43	
Specific IgG antibodies: positive/negative (%)	—	38∗/62	—
Occupational anamnesis: positive/negative (%)		62^∗∗^/38	
Diffusing capacity of lungs for carbon monoxide (DLCO) (%; mean)	88 ± 16	60 ± 16	88 ± 17
Lymphocytes (%; mean) in BALF	37 ± 19	66 ± 19	8 ± 4
Lymphocytes (total number; mean) in BALF	56 ± 51	258 ± 171	5 ± 4
CD3 (%; mean) in BALF	92 ± 6	85 ± 13	81 ± 7
CD3 (total number; mean) in BALF	52 ± 49	231 ± 165	4 ± 3
CD4 (%; mean) in BALF	70 ± 15	43 ± 27	50 ± 11
CD4 (total number; mean) in BALF	41 ± 40	114 ± 112	3 ± 2
CD8 (%; mean) in BALF	22 ± 12	45 ± 29	29 ± 7
CD8 (total number; mean) in BALF	10 ± 11	115 ± 125	2 ± 1
CD4/CD8	5.1 ± 4.5	2.9 ± 4.2	1.8 ± 0.7

∗Cladosporidium, *Thermoactinomyces vulgaris*, *Absidia corymbifera*, *Micropolyspora faeni*, *Actinomyces vulgaris*, *Aspergillus nidulans*, *Aspergillus niger*; ∗∗farmers: 2x, animal feeding: 2x, bird breeders: 2x, textile workers: 1x, chemical industry: 5x, wood workers: 1x; CD3–T cells, CD4–T-helper cells, CD8–cytotoxic T cells; total number–total number of cells per microliter.

**Table 3:** Comparison of sarcoidosis and HP subgroups.

	Sarcoidosis: stage I versus stage II	Sarcoidosis: with versus without Lӧfgren syndrome	HP: stage inflammatory versus fibrotic	Sarcoidosis stage I and II versus inflammatory HP
TREM-1%	80.5/18.4/ *vs* 73.1 /20.2/	79.5/19.2/ *vs* 76.1/21.9/	37.3/38.8/ *vs* 30.8/32.3/	76.7/21.2/ *vs* 26.3/52.8/
Median/IQR/	*P* = 0.005	*P* = 0.1623	*P* = 0.7345	*P* = 0.0002
TREM-1 MFI	44.4/24.5/ *vs* 39.5/24.3/	39.6/22.0/ *vs* 40.9/24.3/	26.9/39.1/ *vs* 24.8/20.7/	40.7/23.3/ *vs* 25.7/38.8/
Median/IQR/	*P* = 0.1985	*P* = 0.6262	*P* = 0.8078	*P* = 0.0395
TREM-2%	37.4/42.1/ *vs* 26.6/37.7/	34.6/36.5/ *vs* 35.0/39.9/	40.1/65.2/ *vs* 45.5/31.4/	34.7/39.0/ *vs* 34.2/42.7/
Median/IQR/	*P* = 0.0648	*P* = 0.7218	*P* = 0.5091	*P* = 0.9143
TREM-2 MFI	8.35/4.0/ *vs* 7.8/3.4/	4.8/3.1/ *vs* 5.0/4.2/	7.0/4.8/ *vs* 7.1/5.3/	5.1/3.9/ *vs* 6.7/4.6/
Median/IQR/	*P* = 0.0654	*P* = 0.7433	*P* = 0.8156	*P* = 0.6845

**Table 4:** TREM-1 and T cells correlation analysis.

TREM-1						
	% *R*	95 CI	*P*	MFI R	95 CI	*P*
Ly total	-0.1163	-0.2651–0.03797	0.1276	-0.1148	-0.2637–0.03946	0.1325
CD3 %	0.2110	0.05935–0.3531	0.0053	0.1908	0.03834–0.3346	0.0119
CD3 total	-0.1029	-0.2524–0.05154	0.1781	-0.1052	-0.2547–0.04918	0.1684
CD4 %	0.2869	0.1395–0.4219	0.0001	0.2156	0.06419–0.3574	0.0044
CD4 total	-0.04006	-0.1925–0.1142	0.6007	-0.06209	-0.2136–0.09237	0.4170
CD8 %	-0.2307	-0.3711–-0.07994	0.0023	-0.1520	-0.2986–0.001655	0.0459
CD8 total	-0.2004	-0.3434–-0.04826	0.0082	-0.1882	-0.3322–-0.03564	0.0132
CD4/CD8	0.2585	0.1092–0.3963	0.0006	0.1811	0.02835 – 0.3257	0.0171

Ly: lymphocytes; total: total number; MFI: mean fluorescence intensity; 95 CI: 95% confidence interval; *R*: Spearman r.

**Table 5:** DLCO, TREM, and T cell subset correlation analysis.

DLCO			
	*R*	95 CI	*P*
Ly total	-0.21166	-0.3506–-0.05178	0.0060
CD3 %	0.04730	-0.1094–0.2017	0.5427
CD3 total	-0.19754	-0.3431–-0.04334	0.0103
CD4 %	0.19429	0.03964–0.3398	0.0116
CD4 total	-0.11665	-0.2679–0.03966	0.1321
CD8 %	-0.15942	-0.3076–-0.003655	0.0390
CD8 total	-0.25418	-0.3954–-0.1035	0.0009
CD4/CD8	0.16987	0.0144–0.3173	0.0277
TREM-1%	0.33172	0.1855–.4636	<0.0001
TREM-1 MFI	0.20269	0.04837–0.3476	0.0084
TREM-2%	0.09735	-0.0594–0.2494	0.2093
TREM-2 MFI	0.03089	-0.1429–0.1687	0.6910

DLCO: diffusing capacity of lungs for carbon monoxide; Ly: lymphocytes; total: total number; MFI: mean fluorescence intensity; 95 CI: 95% confidence interval; *R*: Spearman r.

**Table 6:** Multinomial ordered logit models with dependent variable HRCT.

	(1)	(2)	(3)	(4)
TREM-1%	-0.0280∗∗∗			
	(-3.68)			
CD4^+^ T cells %		-0.0406∗∗∗		
		(-3.86)		
CD8^+^ T cells %			0.0431∗∗∗	
			(3.42)	
DLCO				-0.0358∗∗∗
				(-3.76)
Pseudo *R*^2^	0.033	0.036	0.028	0.035

*t* statistics in parentheses. ∗*p* < 0.05, ∗∗*p* < 0.01, ∗∗∗*p* < 0.001.

**Table 7:** Multinomial ordered logit models with dependent variable HRCT.

	(1)	(2)	(3)	(4)
TREM-1%	-0.0270∗∗			
	(-2.79)			
CD4^+^ T cells %		-0.0398∗∗		
		(-2.95)		
CD8^+^ T cells %			0.0363∗	
			(2.28)	
DLCO				-0.0315∗∗
				(-2.89)
TREM-2%	0.00364	0.000377	-0.000482	-0.00483
	(0.58)	(0.06)	(-0.08)	(-0.82)
Smokers	1.183	1.380∗	1.244	0.900
	(1.90)	(2.15)	(1.96)	(1.40)
Age	0.0130	0.0133	0.0129	0.0112
	(0.89)	(0.91)	(0.89)	(0.75)
Macrophages %	-0.0465	0.00760	0.0128	0.00655
	(-0.41)	(0.07)	(0.12)	(0.06)
Eosinophiles %	-0.217	-0.116	-0.117	-0.159
	(-1.22)	(-0.70)	(-0.70)	(-0.94)
Neutrophils %	-0.0353	0.0330	0.0448	0.0427
	(-0.30)	(0.28)	(0.39)	(0.38)
Lymphocytes %	-0.0348	0.0319	0.0318	0.00281
	(-0.30)	(0.28)	(0.28)	(0.03)
Lymphocytes total	-0.00953	-0.00841	-0.00785	-0.00322
	(-1.88)	(-1.67)	(-1.59)	(-0.63)
sACE	0.642∗	0.771∗	0.802∗	0.574
	(1.98)	(2.38)	(2.45)	(1.74)
Pseudo *R*^2^	0.059	0.061	0.051	0.062

*t* statistics in parentheses. ∗*p* < 0.05, ∗∗*p* < 0.01, ∗∗∗*p* < 0.001.

## Data Availability

The datasets used and/or analysed during the current study are available from the corresponding author on reasonable request.

## Abbreviations

APC: antigen-presenting cells
BALF: bronchoalveolar lavage fluid
CG: control group
DAMPs: damage-associated molecular patterns
DLCO: diffusing capacity of lungs for carbon monoxide
FACS: fluorescence-activated cell sorting
HP: hypersensitivity pneumonitis
HRCT: high-resolution computed tomography
IQR: interquartile range
MFI: mean fluorescence intensity
PAMPs: pathogen-associated molecular patterns
TREM-1: triggering receptor expressed on myeloid cells-1
TREM-2: triggering receptor expressed on myeloid cells-2.
